# ﻿Yeast diversity in traditional fermented foods of ethnic minorities in China, with the descriptions of four new yeast species

**DOI:** 10.3897/imafungus.16.146163

**Published:** 2025-07-09

**Authors:** Shuang Hu, Qi-Yang Zhu, Hai-Yan Zhu, Jun-Yu Liu, Yue Shi, Yan-Jie Qiu, Zhang Wen, Ai-Hua Li, Pei-Jie Han, Feng-Yan Bai

**Affiliations:** 1 State Key Laboratory of Microbial Diversity and Innovative Utilization, Institute of Microbiology, Chinese Academy of Sciences, Beijing 100101, China; 2 College of Life Sciences, University of Chinese Academy of Sciences, Beijing 100049, China; 3 College of Enology and Horticulture, Ningxia University, Yinchuan 750021, China; 4 China General Microbiological Culture Collection Center, Institute of Microbiology, Chinese Academy of Sciences, Beijing 100101, China

**Keywords:** Citizen science initiative, ethnic minority, new yeast species, traditional fermented food, yeast diversity

## Abstract

Fermented foods have been produced by humans since prehistoric times and are consumed worldwide today due to their enhanced nutritional value, taste and flavor, and benefits for human health. Various microorganisms are essential agents responsible for food fermentation and have been extensively studied using both culture-dependent and -independent methods. However, previous research has mainly focused on fermented foods produced on a large scale in urbanized areas. In this study, we collected 255 samples of diverse traditional fermented foods—including alcoholic beverages, amylolytic starters, fruit vinegar, and fermented products of milk, vegetables, cereals, legumes, fish, meat, and other materials—from ethnic minority areas of China through a citizen science initiative for the study of yeast diversity. A total of 516 yeast strains were isolated, and 81 yeast species, including four new species, were identified based on sequence analyses of the D1/D2 domain of the large subunit rRNA gene and the internal transcribed spacer region. The proposals of the new species were further supported by whole-genome average nucleotide identity (ANI) analysis. The dominant species isolated were *Saccharomycescerevisiae*, *Pichiakudriavzevii*, *Wickerhamomycesanomalus*, *Saccharomycopsisfibuligera*, and *Clavisporalusitaniae*. The new species are described as *Blastobotrysguizhouensis***sp. nov.**, *Wickerhamiellashiruii***sp. nov.**, *Trichosporonjiuqu***sp. nov.**, and *Parajaminaeaalba***sp. nov.** This study demonstrates the high yeast diversity in traditional fermented foods of ethnic minorities in China. These yeast resources are of special value for both basic and applied research in the future.

## ﻿Introduction

Food fermentation has been an essential component of human civilization and has been practiced by human beings since prehistoric times. Archaeological evidence of the production of a mixed fermented beverage of rice, honey, and fruit 9,000 years ago in an early Neolithic village of Jiahu in Henan Province, China, has been documented (McGovern et al. 2004). Fermented foods are defined as “foods made through desired microbial growth and enzymatic conversions of food components” (Marco et al. 2021). They can be classified into different groups based on raw materials, including fermented products of cereals, vegetables, legumes, roots/tubers, milk, meat, fish, miscellaneous materials, and alcoholic beverages (Tamang et al. 2016). Fermentation endows foods with many advantages, such as extending shelf life, enhancing food safety, increasing food diversity, eliminating toxic substances and pathogenic microorganisms, promoting digestion and absorption, improving nutritional value, and enriching taste and flavor (Marco et al. 2017; Rezac et al. 2018; Das et al. 2020; Voidarou et al. 2020; Jahn et al. 2023). Today, fermented foods are widely consumed and are integrated into the daily diet, economic development, social activities, cultural customs, and religions of human societies (Tamang et al. 2020; Galimberti et al. 2021).

Fermented foods are the result of microbial activities and interactions in which bacteria, molds, and/or yeasts play indispensable roles. These microorganisms secrete various hydrolytic enzymes that effectively decompose substrates in food matrices and produce a range of bioactive and flavor compounds, thereby enhancing the overall value of the food (Sharma et al. 2020; Tamang et al. 2021). The microbial diversity in fermented foods has been extensively studied using both culture-dependent and -independent methods. Different groups of bacteria and fungi with various functions have been identified in different fermented foods (Tamang et al. 2016; Koricha et al. 2020; Voidarou et al. 2020; Tamang et al. 2021; Tamang 2022; Ghosh et al. 2023; Han et al. 2024). However, previous studies have mainly focused on fermented foods that are commonly consumed or produced on a large industrial scale in cities and urbanized areas. The microbial diversity in fermented foods produced on a regionally limited small scale or by local ethnic minorities remains to be fully revealed.

China has a long history of fermented food production. Diversified fermented foods are produced not only by the main Han but also by every ethnic minority in the country (Jia 2018). China has 55 ethnic minorities, and different ethnic minorities exhibit significant differences in population, regional distribution, religions, historical backgrounds, dietary habits, and socio-economic conditions. Many ethnic minorities utilize unique local resources, such as grapes, wheat, barley, corn, sorghum, rice, oats, buckwheat, barley, and milk, to produce a rich variety of alcoholic beverages and other traditional fermented foods. In contrast to fermented foods in urbanized areas that are usually produced industrially on a large scale using selected commercial starter cultures, the fermented foods of ethnic minorities—especially in remote and economically underdeveloped areas—are usually produced manually on a family or small-mill scale using traditional spontaneous fermentation technologies. The diversity of traditional fermented foods of ethnic minorities is usually better preserved and more closely affiliated with the cultural heritage and history of local ethnic groups. The fermented foods of ethnic minorities are expected to harbor microbes with high species and genetic diversities. These microbes are valuable resources and of special value for the study of the domestication history and evolution of the microbes used for food fermentation.

Yeasts are involved in the production of many fermented foods and play important roles in the fermentation of carbohydrates, the formation of flavor compounds, the usually positive interaction with lactic acid bacteria, the production of tissue-degrading enzymes, and other functional characteristics (Johansen et al. 2019). However, our understanding of the diversity and functions of yeasts in the fermented foods in ethnic minority areas of China is still very limited. The purpose of this study is to investigate the diversity of yeasts in various traditional fermented foods from ethnic minority areas in China, compare the distribution of yeast species in different types of the foods, and collect and preserve indigenous yeast resources for further basic and applied research.

## ﻿Materials and methods

### ﻿Sampling

During 2023, a citizen science initiative was launched with the aim of investigating and collecting traditional fermented foods in ethnic minority areas across China. Qualified volunteers were recruited from different ethnic minority regions and were trained in fermented food sample collection through online communications and courses. A field survey and sampling were also carried out by us in November 2023 in western Sichuan, China. Samples of fermented foods produced without the use of commercial microbial agents such as active dry yeasts, molds, or bacteria as fermentation starters were collected and sent promptly to the laboratory using express mail services. If necessary, ice boxes were used during transportation. Once the samples arrived at our laboratory, they were immediately stored at 4 °C for further processing.

### ﻿Isolation of yeasts

The classic dilution plating method (Duan et al. 2018) was used to isolate yeasts. An aliquot of 5 g of each sample was suspended in 45 mL of sterile water and shaken at 200 rpm for 10 minutes at 25 °C, resulting in a 10^-1^ diluted suspension, which was further diluted (serial dilutions of 10^-2^ to 10^-6^) according to the specific condition of each sample. An aliquot of 200 μL of each dilution was spread on potato dextrose agar (PDA; 20% potato infusion, 2% glucose, and 1.8% agar) and yeast extract peptone dextrose (YPD; 2% glucose, 1% yeast extract, 2% peptone, and 1.8% agar) plates supplemented with 200 μg/mL chloramphenicol. After 3–7 days of incubation at 25 °C, yeast colonies with different appearances (Nascimento et al. 2022) were picked and preserved in 25% glycerol at −80 °C after further purification.

### ﻿Molecular identification of yeasts

DNA of yeast strains was extracted using the method described previously (Wang and Bai 2008). The D1/D2 domain of the large subunit (26S) rRNA gene was amplified and sequenced using the primers NL1 (5’-GCATATCAATAAGCGGAGGAAAAG-3’) and NL4 (5’-GGTCCGTGTTTCAAGACGG-3’) according to Bai et al. (2002). The sequences obtained were compared with those in the GenBank database using the Basic Local Alignment Search Tool (BLAST) (https://blast.ncbi.nlm.nih.gov). Yeast strains with a sequence identity of less than 99% in the D1/D2 domain to the type strains of the most closely related known species were considered as representing potential new species (Vu et al. 2016). The internal transcribed spacer (ITS) region of these strains was then amplified and sequenced using the primers ITS1 (5’-TCCGTAGGTGAACCTGCGG-3’) and ITS4 (5’-TCCTCCGCTTATTGATATGC-3’) (White et al. 1990). The genomes were sequenced on the DNBSEQ-T7 platform using a paired-end sequencing strategy. Raw sequencing data were quality-filtered and adapter-trimmed using *fastp* v. 0.23.2 (Chen et al. 2018), followed by assembly with *SPAdes* v. 3.13.0 (Bankevich et al. 2012). The resulting draft genomes were assessed for assembly quality using *QUAST* v. 5.2.0 (Gurevich et al. 2013). Whole-genome average nucleotide identity (ANI) values were calculated using *PYANI* v. 0.2.13.1 (Pritchard et al. 2016).

### ﻿Phylogenetic analysis

Representative strains of the yeast species identified were selected, and their D1/D2 sequences were deposited in GenBank under the accession numbers PP192688–PP192770. These sequences were aligned using *MAFFT* v. 7 (Katoh and Standley 2013), renamed using *BioEdit* v. 7 (Hall 1999), and used to construct a phylogenetic tree based on Kimura’s two-parameter model using the neighbor-joining algorithm implemented in *MEGA* v. 7 (Kumar et al. 2016; Lachance 2022). Clade stability was estimated using 1,000 bootstrap replicates (Felsenstein 1985). The online open-source tool Interactive Tree of Life (*iTOL*) was used to visualize and annotate phylogenetic trees (Letunic and Bork 2021). For the phylogenetic analysis of strains representing potential new species, the ITS and D1/D2 sequences of these strains were aligned with those of related species in the respective genera and then concatenated to construct a tree using the same method (Suppl. material 1: table S4).

### ﻿Phenotypic characterization of new species

Morphological, physiological, and biochemical characterizations were performed following standard methods described by Kurtzman et al. (2011). Fermentation of sugars and assimilation of carbon and nitrogen compounds were tested in liquid media. Colony and microscopic characteristics were observed on YM agar after 3–5 days of incubation at 25 °C. The potential sexual states of the new species were investigated using PDA, yeast carbon base agar (YCB; 1.17% yeast carbon base and 2% agar), corn meal agar (CMA; 2.5% corn starch and 2% agar), V8 agar (10% V8 juice and 2% agar), and malt extract agar (MEA; 5% malt extract and 2% agar) plates. These media were inoculated with single or pairwise mixed strains, incubated at 25 °C for up to 8 weeks, and observed weekly.

## ﻿Results

### ﻿Diversity and distribution of the fermented foods collected

**Figure 1.**
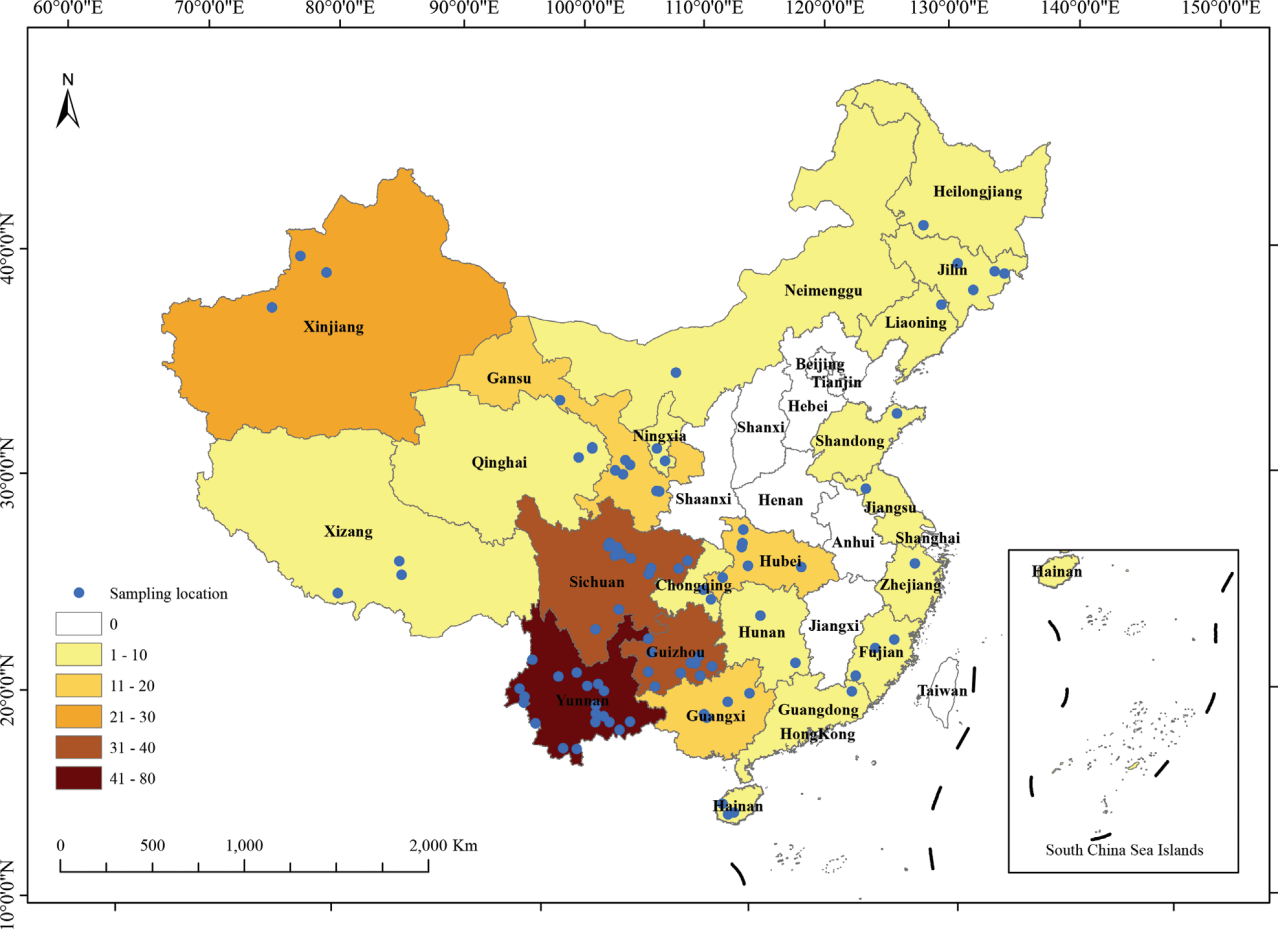
The geographic distribution of the samples of traditional fermented foods of ethnic minorities in China collected in this study. The map was generated by the ArcGIS v. 10.8 software (Esri, Redlands, CA, USA).

In this study, a total of 255 samples were collected from 96 sampling points in 22 provinces of China (Fig. 1). These samples are mainly concentrated in Yunnan (29.80%), Guizhou (12.16%), and Sichuan (12.16%) in Southwest China. The samples covered alcoholic beverages (accounting for 27.1% of the samples collected); amylolytic starters (23.5%); vinegar (7.5%); and fermented products of milk (13.7%), vegetables (11.4%), cereals (6.7%), legumes (5.8%), fish (0.8%), meat (0.4%), and other materials (3.1%) (Suppl. material 1: table S1, Suppl. material 2: fig. S1).

### ﻿The overall yeast diversity in the fermented foods sampled

**Figure 2.**
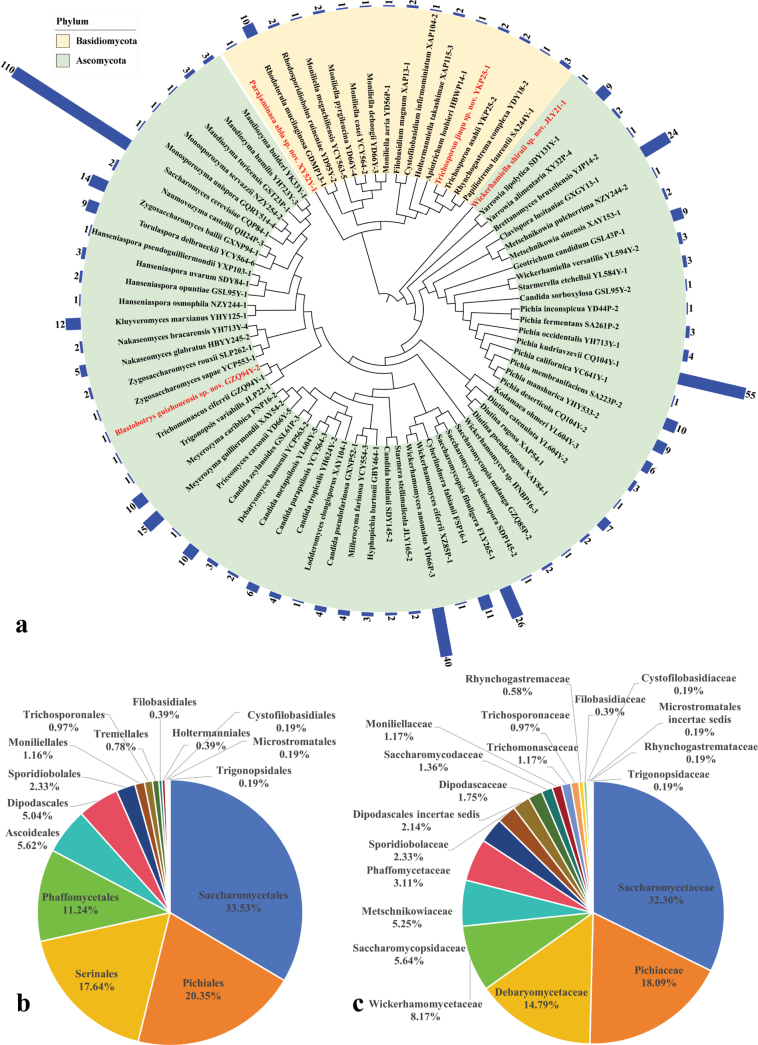
Species statistics. **a** Phylogenetic tree constructed using the neighbor-joining analysis based on the sequences of the 26S rDNA gene D1/D2 domain, showing the relationships of the 65 ascomycetous and 16 basidiomycetous yeast species isolated and identified in this study, along with the number of strains of each species isolated; **b** The proportion of yeast strains isolated belonging to different orders; **c** The proportion of yeast strains isolated belonging to different families.

A total of 516 yeast strains were isolated from the 255 fermented food samples collected. These strains were identified as 81 yeast species, including 65 ascomycetous and 16 basidiomycetous species (Fig. 2a, Suppl. material 1: tables S1, S2). These species belong to eight classes, 15 orders, and 20 families. The dominant classes were *Saccharomycetes* (covering 50.4% of the strains isolated) and *Pichiomycetes* (38.0%); the dominant orders were *Saccharomycetales* (33.5%), *Pichiales* (20.4%), and *Serinales* (17.6%) (Fig. 2b); and the dominant families were *Saccharomycetaceae* (32.0%), *Pichiaceae* (18.1%), and *Debaryomycetaceae* (14.8%) (Fig. 2c). The most frequently isolated species were *Saccharomycescerevisiae* (covering 21.3% of the strains isolated), *Pichiakudriavzevii* (10.7%), *Wickerhamomycesanomalus* (7.8%), *Saccharomycopsisfibuligera* (5.0%), and *Clavisporalusitaniae* (4.7%) (Fig. 2a). *Rhodotorulamucilaginosa* (1.9%) was the most frequently isolated species among the basidiomycetous yeasts isolated (Suppl. material 1: table S2).

### ﻿Distribution of yeast species in fermented foods compared

There were significant differences in the distribution of yeast species in different types of fermented foods studied (Suppl. material 1: table S2). The numbers of yeast strains and species isolated from each type of the fermented foods collected are alcoholic beverages, 115 strains, 24 species; amylolytic starters, 128 strains, 29 species; vinegar products, 37 strains, 13 species; milk products, 98 strains, 34 species; vegetable products, 54 strains, 27 species; cereal products, 32 strains, 17 species; legume products, 27 strains, 17 species; and other products, 13 strains, 11 species. The dominant yeast species isolated from alcoholic beverages were *S.cerevisiae* (occurring in 60.9% of the samples compared in this type, the same as below), *P.kudriavzevii* (20.3%), and *W.anomalus* (18.8%). Among the species from amylolytic starters, *S.cerevisiae* (58.3%), *Sa.fibuligera* (30.0%), and *P.kudriavzevii* (23.3%) exhibited the highest isolation rates. *P.kudriavzevii* (37.9%) was the most dominant species among the yeasts isolated from fermented vegetable products. In vinegar, cereal, and legume products, *S.cerevisiae* was also the most frequently isolated species, and 52.6%, 70.6%, and 26.7% of the samples of the three types collected contained this species, respectively. The fermented milk products contained the highest numbers of species, among which *Clavisporalusitaniae* and *Kluyveromycesmarxianus* exhibited the highest isolation rate, both at 25.7%. More basidiomycetous yeast species were isolated from milk products than from the other types studied. Eleven basidiomycetous yeast species were isolated from the milk products (Suppl. material 1: table S2), accounting for 31.4% of all the species found in the milk products and 68.8% of all the basidiomycetous species isolated from all the traditional fermented foods investigated. In addition, milk products have the highest number of unique species, with 15 yeast species being exclusively isolated from the milk products (Suppl. material 1: table S2). The numbers of the product-type-specific species found in other fermented foods studied are followed by 10 in the amylolytic starters, five in the cereal products, four in the vegetable products, three in the alcoholic beverages, three in the vinegar products, and one in the legume products (Suppl. material 1: table S2). Notably, four yeast species were isolated from two sour fish samples, and seven yeast species were isolated from a sour meat sample (Suppl. material 1: table S2).

### ﻿Phylogenetic analyses of novel yeast species

**Figure 3.**
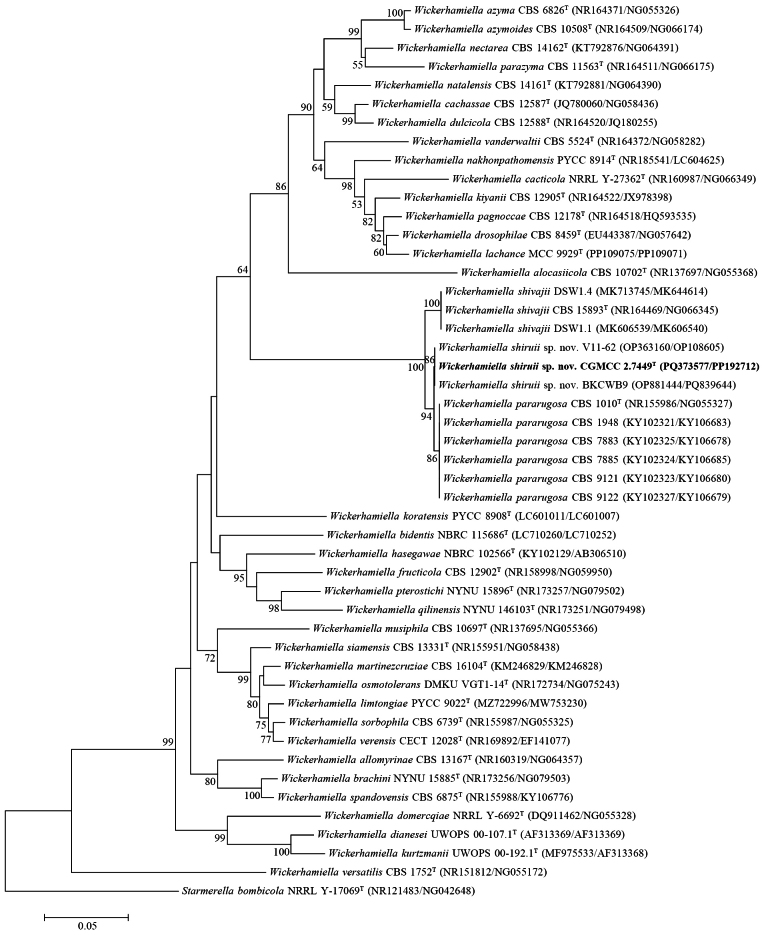
Neighbor‐joining phylogenetic tree showing the phylogenetic position of *W.shiruii* sp. nov. based on the D1/D2 domain and ITS region sequences. *Starmerellabombicola* is used as the outgroup. Bootstrap values above 50% are shown on the branches. Type strains are denoted with the superscript “T.” The bar represents 0.05 substitutions per nucleotide position. The strain isolated in this study is highlighted in bold.

A total of six strains isolated from different fermented foods could not be identified as any known yeast species because of their significant sequence differences from closely related described species in the D1/D2 and ITS regions. Strain CGMCC 2.7449 (original number JLY21-1), isolated from a rice wine sample, possesses similar sequences (only one base mismatch) in the D1/D2 domain to strains V11-62, V11-64, V11-70, V12-75, and V11-63NL isolated from ethnic Slovak cow-milk bryndza; Cd2 from Czech Republic semi-hard cheese; LA118 and LA206 from Italian cheese; NM 2b from Mexican sour maize gruel; VTT C-04522 from Finnish industrial malting ecosystem; S3L3 from Algerian fermented butter; BKCWB9 from Turkish raw milk; and CBS 2275 from Dutch rancid butter (Suppl. material 2: fig. S2). The result of a BLAST search through GenBank against the ITS sequence of strain CGMCC 2.7449 showed that this strain exhibited only one base mismatch in the ITS region with strains V11-62 and BKCWB9 mentioned above; strains AUMC 13713 and AUMC 13734 isolated from Egyptian fermented dairy products; strain Cli6 from an unknown source in Iran; and strain CNRMA10.115 from a clinical source in France (Suppl. material 2: fig. S3). The sequence comparisons showed that strain CGMCC 2.7449 and the other 17 strains mentioned above are conspecific, but most of the foreign strains were previously identified as *Wickerhamiellapararugosa*. In fact, strain CGMCC 2.7449 differs from *W.pararugosa* CBS 1010ᵀ by six (1.2%, five substitutions and one gap) and 12 (3.3%, seven substitutions and five gaps) mismatches and from another closely related species, *W.shivajii* CBS 15893ᵀ, by 13 (2.6%, 11 substitutions and two gaps) and 14 (3.8%, six substitutions and eight gaps) mismatches in the D1/D2 domain and ITS region, respectively (Fig. 3). The results suggest that strain CGMCC 2.7449 and the other 17 foreign strains represent a novel species, for which the name *Wickerhamiellashiruii* sp. nov. is proposed.

**Figure 4.**
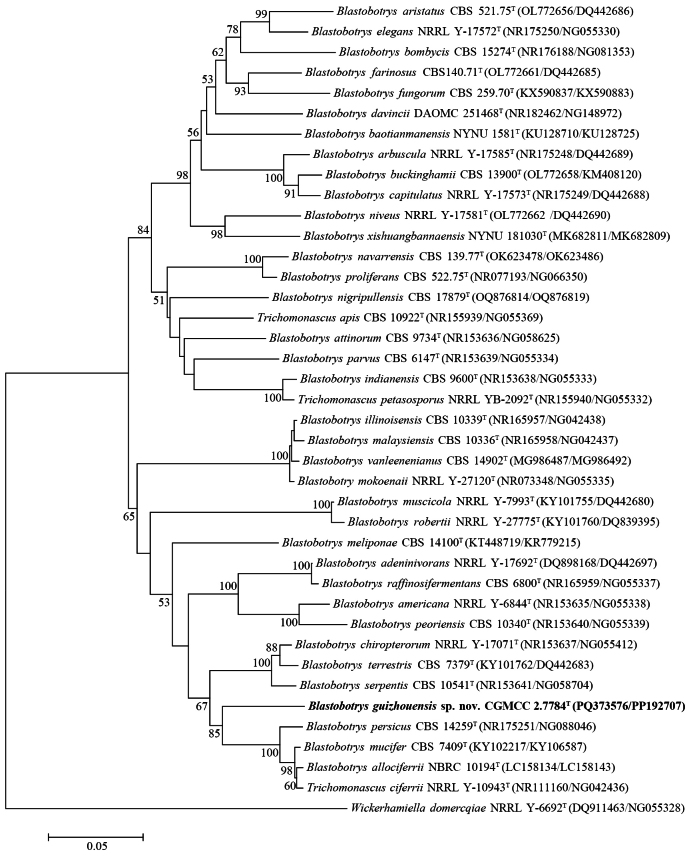
Neighbor‐joining phylogenetic tree showing the phylogenetic position of *B.guizhouensis* sp. nov. based on the D1/D2 domain and ITS region sequences. *Wickerhamielladomercqiae* is used as the outgroup. Bootstrap values above 50% are shown on the branches. Type strains are denoted with the superscript “T.” The bar represents 0.05 substitutions per nucleotide position. The strain isolated in this study is highlighted in bold.

Strain CGMCC 2.7784 (original number GZQ94Y-2) isolated from a jiuqu sample is located in a branch basal to *Blastobotryspersicus*, *B.allociferrii*, *B.mucifer*, and *Trichomonascusciferrii* in the genus *Blastobotrys* with strong bootstrap support (Fig. 4). It differs from the closest species, *B.persicus* CBS 14259ᵀ, by 28 (5.0%, 26 substitutions and two gaps) and 107 (17.9%, 53 substitutions and 54 gaps) mismatches in the D1/D2 domain and ITS region, respectively, strongly suggesting that strain CGMCC 2.7784 represents a novel species, for which the name *Blastobotrysguizhouensis* sp. nov. is proposed.

**Figure 5.**
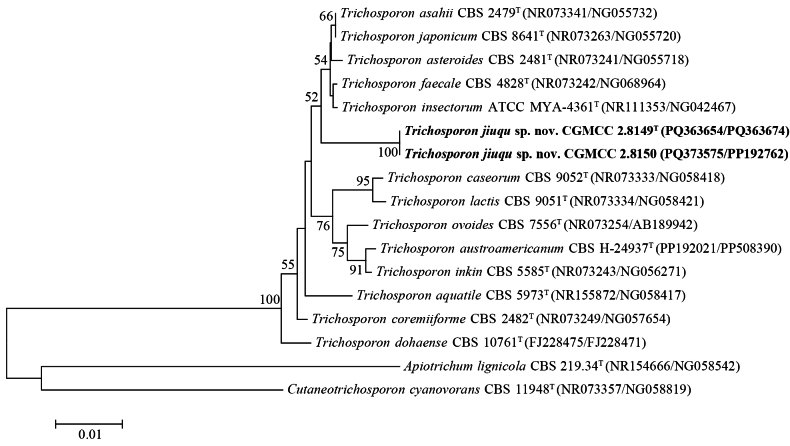
Neighbor‐joining phylogenetic tree showing the phylogenetic position of *T.jiuqu* sp. nov. based on the D1/D2 domain and ITS region sequences. *Apiotrichumlignicola* and *Cutaneotrichosporoncyanovorans* are used as outgroups. Bootstrap values above 50% are shown on the branches. Type strains are denoted with the superscript “T.” The bar represents 0.01 substitutions per nucleotide position. The strains isolated in this study are highlighted in bold.

Two strains, CGMCC 2.8149 (original number YKP15-1) and CGMCC 2.8150 (original number YKP25-1), isolated from different *jiuqu* samples have the same D1/D2 and ITS sequences, indicating that they belong to the same species. They formed an independent branch in the *Trichosporon* clade and were closely related to a subclade containing *T.asahii*, *T.asteroides*, *T.japonicum*, *T.faecale*, and *T.insectorum*, but the bootstrap value is low (Fig. 5). Strains CGMCC 2.8149 and CGMCC 2.8150 differ from the five known species by eight (1.4%) to 14 (2.4%) mismatches in the D1/D2 domain and by three (0.6%) to four (0.8%) mismatches in the ITS region. The result suggests that the two strains represent a novel species, for which the name *Trichosporonjiuqu* sp. nov. is proposed.

**Figure 6.**
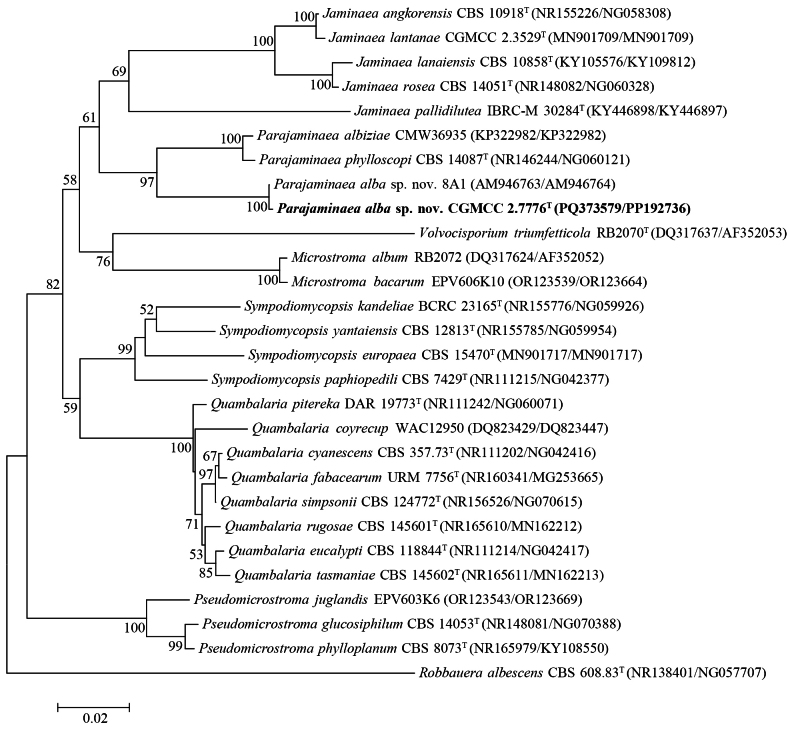
Neighbor‐joining phylogenetic tree showing the phylogenetic position of *P.alba* sp. nov. based on the D1/D2 domain and ITS region sequences. *Robbaueraalbescens* is used as the outgroup. Bootstrap values above 50% are shown on the branches. Type strains are denoted with the superscript “T.” The bar represents 0.02 substitutions per nucleotide position. The strain isolated in this study is highlighted in bold.

Strain CGMCC 2.7776 (original number XY52Y-1), isolated from yogurt, forms a branch together with strain 8A1, isolated from a plant in Rawatbhata, India. The two strains have only one base difference in each of the D1/D2 and ITS regions, indicating that they are conspecific. They are closely related to the two known species of the genus *Parajaminaea* with strong bootstrap support in the order *Microstromatales*. They differ from *P.phylloscopi* CBS 14087ᵀ by 20 (3.6%, 19 substitutions and one gap) and 64 (10.5%, 48 substitutions and 16 gaps) mismatches and from *P.albiziae* CMW36935 by 33 (5.8%, 32 substitutions and one gap) and 64 (10.5%, 48 substitutions and 16 gaps) mismatches in the D1/D2 domain and ITS region, respectively (Fig. 6). Therefore, we assign strains CGMCC 2.7776 and 8A1 to the genus *Parajaminaea* and propose a novel species, *Parajaminaeaalba* sp. nov., for them.

### ﻿Genome sequence analysis

We sequenced the genomes of the types of the four proposed new species, *W.shiruii*, *B.guizhouensis*, *T.jiuqu*, and *P.alba* (Table 1), and their DDBJ/ENA/GenBank whole genome shotgun project accession numbers are listed in Suppl. material 1: table S4. The tetra analysis linked *W.shiruii* to a subclade containing *W.pararugosa* and *W.shivajii* (Suppl. material 2: fig. S4). The close relationship among these three species is consistent with that depicted in the tree constructed from the ITS and D1/D2 sequences (Fig. 3). The ANI values between the *Wickerhamiella* species range from 82.1% to 95.5% (Suppl. material 1: table S5). *W.shiruii* and *W.shivajii* exhibit an ANI value of 92.2%, confirming their separation as distinct species. The 95.5% ANI value between *W.shiruii* and *W.pararugosa* is at the threshold of 95%, which is considered a preliminary criterion for yeast species delineation in some studies (Cortimiglia et al. 2024; Fay et al. 2024). However, interspecific ANI values above 95% have also been observed between some well-separated yeast species, such as *Metschnikowiadrakensbergensis* and *Metschnikowiaproteae* (95.2%) (Lachance et al. 2020), and *Cryptococcusgattii* and *Cryptococcusbacillisporus* (95.2%) (Libkind et al. 2020). The ANI values (>99.7%) between the five *W.pararugosa* strains with different origins compared (Suppl. material 1: table S5, Suppl. material 2: fig. S4) suggest intra-specific genome sequence homogeneity of *W.pararugosa* strains. The nearly identical D1/D2 sequences of the 14 *W.shiruii* strains from different countries (Suppl. material 2: fig. S2) imply intra-specific sequence homogeneity of *W.shiruii*. The highly conserved intraspecific nature and clear interspecific divergence between *W.shiruii* and *W.pararugosa* in DNA barcoding regions and whole genome sequences support the proposal of *W.shiruii* as a new species. Furthermore, *W.shiruii* differs phenotypically from *W.pararugosa* in its ability to grow in inulin and ethylamine, on 50% glucose, and at 40 °C and 42 °C (Suppl. material 1: table S3).

**Table 1.** Genome sequence data of the type strains of four novel species.

Strain	Accession number	Sequence depth	Contigs (>500 bp)	Genome size (Mb)	Largest contig (Mb)	N50 (kb)	GC content
CGMCC 2.7449	JBMKKW000000000	271×	76	9.1	1.9	767.7	50.0%
CGMCC 2.7784	JBMKKV000000000	159×	115	21.3	1.0	698.8	44.5%
CGMCC 2.8149	JBMKKY000000000	115×	102	24.9	1.5	696.2	58.8%
CGMCC 2.7776	JBMKKX000000000	115×	52	16.3	2.7	815.1	55.2%

The tetra analysis linked *B.guizhouensis* to a subclade containing *B.niveus*, *B.parvus*, *B.proliferans*, and *Trichomonascusapis* (Suppl. material 2: fig. S5), which is inconsistent with the tree constructed from the ITS and D1/D2 sequences (Fig. 4). The ANI values between *B.guizhouensis* and the other *Blastobotrys* species ranged from 82% to 90% (Suppl. material 1: table S5), supporting the proposal of *B.guizhouensis* as a new species. The ANI values between the *Blastobotrys* species ranged from 81% to 98%, with the highest values observed between *B.capitulatus* and *Trichomonascusciferrii* (98.1%) and between *B.raffinosifermentans* and *B.adeninivorans* (98.2%) (Suppl. material 1: table S5), suggesting that each of these species pairs may belong to the same species.

The tetra analysis of eight *Trichosporon* species with available genome sequences indicates that *T.jiuqu* forms a relatively independent branch (Suppl. material 2: fig. S6), consistent with that resolved in the rDNA tree (Fig. 5). The ANI values between the *Trichosporon* species compared range from 84% to 90%, and the ANI values between *T.jiuqu* and the other *Trichosporon* species range from 84.7% to 87.3% (Suppl. material 1: table S5), suggesting that *T.jiuqu* is a well-separated species. We sequenced the genome of *P.alba* strain CGMCC 2.7776, but the genome data of the other two known *Parajaminaea* species are not available for the calculation of ANI values.

### ﻿Taxonomic descriptions

#### ﻿ Blastobotrys guizhouensis

Taxon classificationAnimaliaSaccharomycetalesTrichomonascaceae

S. Hu, Q.Y. Zhu & F.Y. Bai sp. nov.

6FE1DFDB-E454-5343-BBAD-1E9AF439B702

Fungal Names: FN 572190

##### Etymology.

gui.zhou.en’sis. N.L. gen. masc. adj. *guizhouensis* of Guizhou, referring to the geographic origin of the type strain of this species.

##### Type.

**CHINA** • Guizhou Province, Qiannan Buyi and Miao Autonomous Prefecture, Anlong County, 27.0319°N, 107.5333°E, from gray jiuqu made by Buyi nationality, August 2023, Q.Y. Zhu. The holotype CGMCC 2.7784 (original number GZQ94Y-2) has been preserved in a metabolically inactive state in the China General Microbiological Culture Collection Center (CGMCC), Beijing, China. An ex­type culture has been deposited in the Japan Collection of Microorganisms (JCM), Koyadai, Japan, as JCM 36892. GenBank accessions: ITS-PQ373576 and LSU-PP192707. The raw genome data of CGMCC 2.7784 has been deposited in GenBank under the BioSample accession numbers SAMN47538173.

##### Culture characteristics.

**Figure 7.**
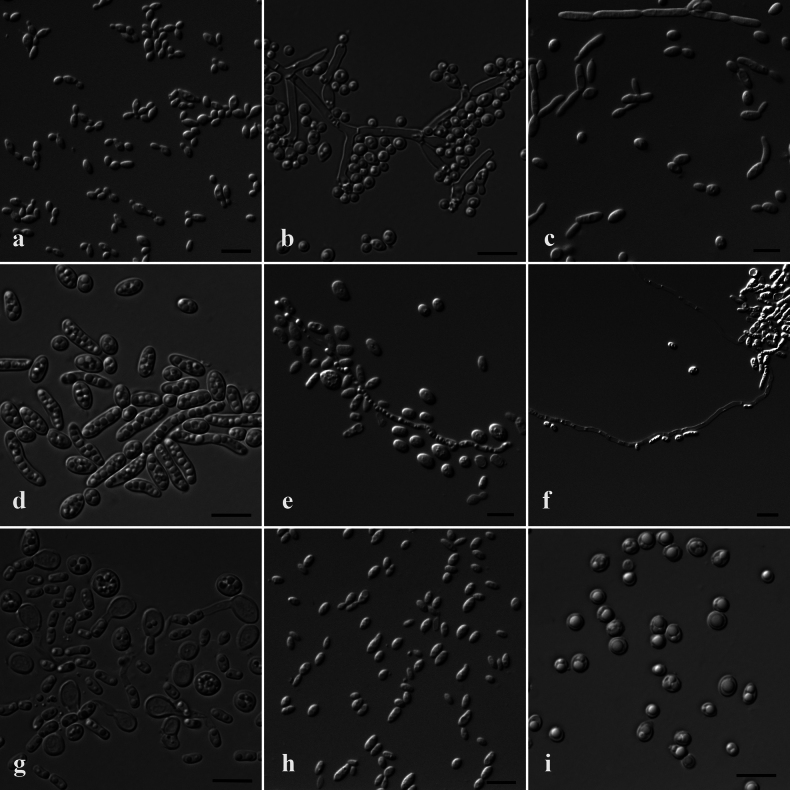
Cellular morphology of the four novel species. *Blastobotrysguizhouensis* sp. nov. CGMCC 2.7784^T^. **a** Vegetative cells on YM agar at 25 °C after 3 days; **b** Conidiophores and conidia on PDA agar at 25 °C after 2 weeks. *Wickerhamiellashiruii* sp. nov. CGMCC 2.7449^T^: **c** Vegetative cells and pseudohyphae on YM agar at 25 °C after 5 days; **d** Containing many oil droplet cells on YCB agar at 25 °C after 2 weeks. *Trichosporonjiuqu* sp. nov. CGMCC 2.8149^T^: **e** Vegetative cells on YM agar at 25 °C after 3 days; **f** True hyphae on MEA agar at 25 °C after 2 weeks; **g** Blastoconidia on YCB agar at 25 °C after 2 weeks. *Parajaminaeaalba* sp. nov. CGMCC 2.7776^T^: **h** Vegetative cells on YM agar at 25 °C after 3 days. **i** Containing oil droplet cells on PDA agar at 25 °C after 2 weeks. Scale bars: 10 µm.

After growth on YM agar for 3 days at 25 °C, colonies are light yellow, butyrous, circular, and slightly raised, with a smooth surface and entire margins. Cells are ovoid to ellipsoidal, 2–3 × 3–5 µm. Budding is multilateral (Fig. 7a). After 1 month in YM broth at 25 °C, sediment is present, but pellicle is absent. After growth on PDA agar for 2 weeks at 25 °C, septate hyphae with a diameter of about 2.2 µm are formed. Conidiophores are formed on the hyphae and bare spherical or short-ovoid blastoconidia (Fig. 7b). The sexual state was not observed.

Physiological characteristics of the new species are listed in Table 2.

**Table 2.** Physiological and biochemical characteristics of the four novel yeast species.

		*W.shiruii*	*B.guizhouensis*	*T.jiuqu*	*P.alba*
Fermentation	D-Glucose	−	D	−	−
	D-Galactose	−	W	−	−
	Sucrose	−	−	−	−
	Maltose	−	−	−	−
	Lactose	−	−	−	−
	Raffinose	−	−	−	−
	Trehalose	−	−	−	−
Assimilation of carbon compounds	D-glucose	+	+	W/+	+
	D-galactose	+	+	W/+	D
	L-sorbose	+	D	−	W
	Sucrose	−	−	W/+	D
	Maltose	−	+	W/+	+
	Cellobiose	−	D	W/+	W
	Trehalose	−	+	W/+	−
	Lactose	−	−	W/+	S
	Melibiose	−	−	−	−
	Raffinose	−	−	−	W
	Melezitose	−	−	−	+
	Inulin	+	−	+	+
	Soluble starch	−	−	W/+	−
	D-Xylose	−	S	W/+	S
	L-Arabinose	−	S	−	W
	D-Arabinose	−	−	−	−
	D-Ribose	−	D	W/+	W
	L-Rhamnose	−	W	−	−
	D-Glucosamine	−	W	−	−
	Methanol	−	−	−	−
	Ethanol	S	S	W/+	+
	Glycerol	S/+	S	W/+	+
	Erythritol	−	D	W/+	+
	Ribitol	−	D	−	−
	Galactitol	−	D	−	−
	D-Mannitol	D	S	−	S
	D-Glucitol	+	D	−	W
	α-methyl-D-Glucoside	−	−	W/+	W
	Salicin	−	S	W/+	−
	D-Glucuronic acid	−	−	−	−
	DL-Lactic acid	S	−	W/+	−
	Succinic acid	S/+	−	−	−
	Sodium citrate Dihydrate	−	−	W/−	−
	Inositol	−	W	−	−
	Hexadecane	−	−	−	−
	N-acetyl-D-Glucosamine	−	+	+	−
	Xylitol	+	W	−	+
Assimilation of nitrogen compounds	Potassium nitrate	−	−	−	S
	Sodium nitrite	−	−	−	−
	Ethylamine	W/−	W	W/+	−
	Cadaverine	D	W	W/+	−
	L-Lysine	D	W	W/+	W
	Ammonium sulfate	+	S	W	D
Other tests	Vitamin-free	W	W	−	D
	10% NaCl	W	+	−	W
	50% glucose	W/+	+	−	+
	60% glucose	W/−	+	−	W
	Urease activity	−	−	+	+
	Diazonium Blue B	−	−	+	+
	Starch formation	−	−	−	−
Temperature tolerance	30 °C	+	+	+	+
	37 °C	+	−	+	−
	40 °C	W/+	−	W	−
	42 °C	W	−	−	−

Note: +, positive; −, negative; D, delayed positive; S, slow positive; W, weakly positive.

##### Notes.

Physiologically, *B.guizhouensis* differs from its close relative *B.persicus* in the fermentation reactions of glucose and galactose; assimilation reactions of sucrose, melibiose, raffinose, melezitose, ribose, mannitol, DL-lactic acid, inositol, hexadecane, ethylamine, cadaverine, and L-lysine; and growth on 60% glucose and at 37–42 °C (Suppl. material 1: table S3). A total of 35 species are currently accepted in the genus *Blastobotrys* (Visagie et al. 2024), which exist in diversified environments, including cave soil, indoor air, paintings, rotten wood, insect guts, and animal livers (Kurtzman 2007; Middelhoven and Kurtzman 2007; Nouri et al. 2018; Chai et al. 2020; Visagie et al. 2024). Only two *Blastobotrys* species have been found from foods. *B.meliponae* was isolated from honey in Brazil (Crous et al. 2016). *B.adeninivorans* was found to be a dominant species in the fermentation process of different tea products in different regions of China (Li et al. 2018; Long et al. 2023; Wang et al. 2023). This is the first time to find a species of this genus from amylolytic starters. It is worthy of further study on the source of *B.guizhouensis* and its function in jiuqu.

##### Distribution.

China.

#### ﻿ Wickerhamiella shiruii

Taxon classificationAnimaliaSaccharomycetalesTrichomonascaceae

S. Hu, Q.Y. Zhu & F.Y. Bai sp. nov.

091A715E-7211-594E-8537-900192697F07

Fungal Names: FN 572191

##### Etymology.

shi.ru’ii. N.L. gen. sing. n. *shiruii* of Shiru, referring to Shiru Jia at Tianjin University of Science and Technology in recognition of his contributions to the research on traditional fermented foods in China.

##### Type.

**CHINA** • Jilin Province, Yanbian Korean Autonomous Prefecture, 43.0663°N, 128.8593°E, from rice wine made by Korean nationality, June 2023, Q.Y. Zhu. The holotype CGMCC 2.7449 (original number JLY21-1) has been preserved in a metabolically inactive state in the China General Microbiological Culture Collection Center (CGMCC), Beijing, China. An ex­type culture has been deposited in the Japan Collection of Microorganisms (JCM), Koyadai, Japan, as JCM 36917. GenBank accessions: ITS-PQ373577 and LSU-PP192712. The raw genome data of CGMCC 2.7449 has been deposited in GenBank under the BioSample accession numbers SAMN47538174.

##### Culture characteristics.

After growth on YM agar for 5 days at 25 °C, colonies are white and villous, with a rough surface and irregular margins. Cells are circular to ovoid, 3–3.5 × 3.5–6 µm. Budding is multilateral. Pseudohyphae consist of elongated cells that are formed (Fig. 7c). After 4 weeks in YM broth at 25 °C, sediment and pellicle are formed. After growth on YCB/PDA agar for 2 weeks at 25 °C, oval or elongated cells (3.5–5 × 4.5–24 µm) and probably oil droplets are formed (Fig. 7d). The sexual stage was not detected.

Physiological characteristics of the new species are listed in Table 2.

##### Notes.

Physiologically, *W.shiruii* differs from its closest relative, *W.pararugosa*, in the utilization of inulin and ethylamine and growth on 50% glucose in vitamin­free medium and 10% NaCl plus 5% glucose medium and at 40–42 °C (Suppl. material 1: table S3). A total of 47 species are currently accepted in the genus *Wickerhamiella*; the ecological distribution of them is mostly associated with flowers, insects, fruits, and foliage, with some instances of isolation from anthropic environments such as wineries, alcoholic beverages, and even human sources (Liu et al. 2016; Belloch et al. 2020; Sakpuntoon et al. 2020). *Wickerhamiella* species may occur in specific regions, such as the Neotropics and Asia, or exhibit cosmopolitan distribution (Goncalves et al. 2020). It seems that *W.shiruii* commonly exists in specific alcoholic beverages and fermented dairy products. Among the 17 non-Chinese strains that are conspecific with the type strain CGMCC 2.7449 of the new species isolated in this study, 13 were isolated from various fermented dairy products in different countries located on different continents, suggesting a worldwide distribution of the new species in fermented foods. A prominent feature of *W.shiruii* is its ability to grow at 42 °C, which is a rare trait in this genus (Avchar et al. 2019). In addition, many oil droplet-like structures were observed in the cells of *W.shiruii*, implying the potential of the new species as an oleaginous yeast to produce micro-biodiesel (Salvador López et al. 2022; Zhao et al. 2023).

##### Distribution.

China, Slovakia, Egypt, Turkey, Holland, the Czech Republic, Iran, Australia, Mexico, Algeria, Finland, and Italy.

#### ﻿ Trichosporon jiuqu

Taxon classificationAnimaliaTrichosporonalesTrichosporonaceae

S. Hu, Q.Y. Zhu & F.Y. B﻿﻿﻿ai sp. nov.

509F52C5-7925-5295-AEDB-8E8BD4CA9D05

Fungal Names: FN 572192

##### Etymology.

jiu.qu. N.L. gen. neut. n. *jiuqu* of a Chinese traditional fermentation starter, referring to the isolation substrate of the type strain.

##### Type.

**CHINA** • Yunnan Province, Kunming City, 25.0383°N, 102.7221°E, from jiuqu made by the Li nationality, June 2023, Q.Y. Zhu. The holotype CGMCC 2.8149 (original number YKP15-1) has been preserved in a metabolically inactive state in the China General Microbiological Culture Collection Center (CGMCC), Beijing, China. An ex­type culture has been deposited in the Japan Collection of Microorganisms (JCM), Koyadai, Japan, as JCM 36915. GenBank accessions: ITS-PQ363654 and LSU-PQ363674. The raw genome data of CGMCC 2.8149 has been deposited in GenBank under the BioSample accession numbers SAMN47538175.

##### Culture characteristics.

After growth on YM agar for 5 days at 25 °C, colonies are white and butyrous, with a glossy, moist surface and entire margins. Cells are ovoid to ellipsoidal, 2–4 × 4–6 µm (Fig. 7e). Septate hyphae and arthroconidia (2–2.5 × 3–6 µm) are present after growth on MEA/YCB/PDA agar for 2 weeks at 25 °C (Fig. 7f, g). After 4 weeks in YM broth at 25 °C, sediment and pellicle were observed. Sexual structures were not observed.

Physiological characteristics of the new species are listed in Table 2.

##### Notes.

*T.jiuqu* sp. nov. can be clearly distinguished from its close relatives in physiological and biochemical characteristics (Suppl. material 1: table S3). *T.jiuqu* is the third species found exclusively in fermented foods in this genus, apart from *T.caseorum* and *T.lactis*. In fact, the emergence of other *Trichosporon* species in the brewing environment has also been reported. For example, *T.coremiiforme*, *T.insectorum*, and *T.inkin* were reported to exist in the pit mud and zaopei of strong-flavor Baijiu production (Pu and Yan 2022; Xu et al. 2022), and *T.asteroides* was reported to be related to the formation of volatile compounds in Hakka rice wine (Wang et al. 2024). Half of the species in this genus can cause internal and external infections in the human body (Takashima et al. 2019; Takashima and Sugita 2019), so the functionality and safety of *T.jiuqu* require further research.

##### Distribution.

China.

#### ﻿ Parajaminaea alba

Taxon classificationAnimaliaMicrostromatalesTrichosporonaceae

S. Hu, Q.Y. Zhu & F.Y. Bai sp. nov.

09A5D949-7F40-5ECA-8593-56FBDF7504D4

Fungal Names: FN 572193

##### Etymology.

al’ba. L. gen. fem. adj. *alba*, white, referring to the color of colonies.

##### Type.

**CHINA** • Xinjiang Uygur Autonomous Region, Ili Kazakh Autonomous Prefecture, Xinyuan County, 43.3928°N, 83.2205°E, from yogurt made by Kazakh nationality, August 2023, S. Hu. The holotype CGMCC 2.7776 (original number XY52Y-1) has been preserved in a metabolically inactive state in the China General Microbiological Culture Collection Center (CGMCC), Beijing, China. An ex­type culture has been deposited in the Japan Collection of Microorganisms (JCM), Koyadai, Japan, as JCM 36899. GenBank accessions: ITS-PQ373579 and LSU-PP192736. The raw genome data of CGMCC 2.7776 has been deposited in GenBank under the BioSample accession numbers SAMN47538176.

##### Culture characteristics.

After growth on YM agar for 5 days at 25 °C, colonies are white, butyrous, and slightly raised, with a smooth, moist surface and entire margins. Cells are ellipsoidal to fusiform, 2–3 × 3–6 µm. Budding is monopolar, and pseudohyphae or true hyphae are not formed (Fig. 7h). After 4 weeks in YM broth at 25 °C, sediment and pellicle are present. After growth on PDA agar for 2 weeks at 25 °C, oil droplet-like structures were observed within the cells (Fig. 7i). Sexual structures were not observed.

Physiological characteristics of the new species are listed in Table 2.

##### Notes.

The genus *Parajaminaea* was proposed in 2017 to accommodate *P.albizae* (*Microstromaalbiziae*) and *P.phylloscopi* (*Jaminaeaphylloscopi*) (Kijpornyongpan and Aime 2017). The authors assigned strain 8A1 to this genus as representing a distinct undescribed species, which is formally described here as *P.alba* together with strain CGMCC 2.7776. Strain 8A1 was isolated from the plant, implying different ecological distributions of the new species. *P.phylloscopi* was isolated from a trans-Saharan migratory bird in Italy and can grow at 44 °C and at pH 2.5 (Francesca et al. 2016), and *P.albiziae* is a foliar pathogen on woody plant species of the genus *Albizia* (Kijpornyongpan and Aime 2017), suggesting ecological diversity of the genus *Parajaminaea*. *P.alba* differs from *P.phylloscopi* phenotypically in the assimilation reactions of trehalose, starch soluble, D-arabinose, ribitol, DL-lactic acid, succinic acid, and sodium nitrite; the ability to grow at 37 °C; and the Diazonium Blue B reaction (Suppl. material 1: table S3). It is worth testing whether the abundant droplet-like structures in the cells of *P.alba*, as shown in Fig. 7i, are formed by oil.

##### Distribution.

China, India.

## ﻿Discussion

In this study, we collected a large set of samples of diversified traditional fermented foods from ethnic minority areas in China through a citizen science initiative combined with on-site investigation and sampling by the authors. The yeast diversity in the fermented foods collected was investigated using conventional culture-dependent methods, and a total of 81 yeast species, including four new species, were identified. We revealed the high diversity of yeasts in traditional fermented foods in ethnic minority areas in China and call for further studies on their functions and biotechnological potentials.

The distribution and diversity of traditional fermented foods of ethnic minorities in China have been extensively investigated by researchers from different regions of the country (Jia 2018). Most of these foods, still produced using traditional handicraft techniques, are typically found in remote areas. We successfully collected many such samples of wide national origin through a citizen science initiative. Citizen science refers to scientific programs overseen by professional scientists in which data are collected and/or analyzed by non-professional volunteers and then used to advance scientific knowledge (McKinley et al. 2017; Heigl et al. 2019; MacPhail and Colla 2020). This approach has been applied in multiple research fields, including ecological environmental protection (Winton et al. 2023), invasive species monitoring (Pocock et al. 2024), endangered species discovery (Santangeli et al. 2020), climate change documentation (Dawson et al. 2020), biodiversity exploration (Wangyal et al. 2022), epidemiological investigation (Froeling et al. 2024), and the promotion of human physical and mental health (Treitler et al. 2023). Recently in China, citizen science was used to collect grain samples for investigating *Fusarium* diversity associated with diseased cereals (Han et al. 2023). Our study demonstrates that citizen science is also a promising method for collecting fermented food samples from remote regions, made feasible by China’s extensive express mail networks.

Yeast diversity in various traditional fermented foods of ethnic minorities in China has been reported previously, such as *musalais* fermented from boiled grape juice in Xinjiang (Zhu et al. 2012); *koumiss* from mare’s milk in Inner Mongolia, Tibet, and Xinjiang (Mu et al. 2012); *suancai* from vegetables in Guizhou, Sichuan, and Yunnan (Liu et al. 2021b); wine from local grapes in Guizhou (Wang et al. 2019; Liu et al. 2021a); and soy sauce from soybeans or black beans (Wang et al. 2022). However, a comprehensive study across different fermented food types was lacking. Our findings provide improved understanding of the diversity and distribution of yeasts in these foods. Although high-throughput sequencing is commonly used for investigating microbial diversity in fermented food (Gounari et al. 2023; Luo et al. 2023; Zhao et al. 2024), previous studies have shown that unculturable microbes are usually absent or rare in such environments. We opted for conventional culture-dependent methods not only for precise species identification but also to obtain pure cultures for future research. Yeasts in fermented foods are likely to have undergone artificial selection through intentional or unintentional domestication over hundreds or thousands of years, which may confer enhanced fermentation or stress tolerance. Indeed, many commercial yeast strains used for bioethanol production were developed from *Saccharomycescerevisiae* strains originally involved in traditional food fermentation, as shown by our prior phylogenomic analysis (Duan et al. 2018). Apart from *S.cerevisiae*, other species isolated in this study, including *Kluyveromycesmarxianus*, *Pichiakudriavzevii*, *Yarrowialipolytica*, and *Zygosaccharomycesbailii* (Suppl. material 1: table S2), should be further screened for biotechnological potential (Binati et al. 2021; Geijer et al. 2022).

The yeast strains—especially *S.cerevisiae*—isolated in this study are also valuable for studying the domestication and evolutionary history of food-fermenting yeasts. For example, the traditional alcoholic beverage *Zajiu* collected in this study (Suppl. material 1: table S1) is produced by different ethnic minorities across China (Jia 2018). *Zajiu* is brewed in pottery jars and consumed through bamboo tubes, resembling the production and drinking practices of ancient Egyptian beer or wine, as illustrated in Egyptian murals (McGovern 2009). The traditional production techniques of *Zajiu* and similar beverages (e.g., *Gan-Gan Jiu*) in remote areas may represent living fossils of ancient fermentation technology. It would be interesting to analyze the evolutionary relationships between *S.cerevisiae* strains from *Zajiu* and those used in other alcoholic beverages using phylogenomics (Bai et al. 2022). *S.cerevisiae* was also dominant in fermented milk products (Suppl. material 1: tables S1, S2). Our previous work showed that dairy-associated *S.cerevisiae* strains represent a distinct lineage with adaptive genomic features (Duan et al. 2019). The roles and evolutionary relationships of *S.cerevisiae* strains found unexpectedly in vinegar and fermented legumes (Suppl. material 1: tables S1, S2) remain to be explored.

This study also highlights potential food safety concerns. Several pathogenic or opportunistic yeast species were detected in the samples, including *P.kudriavzevii* (= *Candidakrusei*) (Zheng et al. 2024), *Cyberlindnerafabianii* (= *Candidafabianii*) (Arastehfar et al. 2019), *Candidaparapsilosis* (Govrins and Lass-Flörl 2023), *Candidatropicalis* (Keighley et al. 2024), *Candidazeylanoides* (Shokri 2014), *Clavisporalusitaniae* (= *Candidalusitaniae*) (Rojas et al. 2024), *Diutinarugosa* (= *Candidarugosa*) (Ming et al. 2019), *Kodamaeaohmeri* (Ioannou and Papakitsou 2020), *Nakaseomycesglabratus* (= *Candidaglabrata*) (Takashima and Sugita 2022), and *Trichosporonasahii* (Commenges et al. 2024). Notably, *C.parapsilosis* was isolated from 80% of the *milk fan* samples—fermented milk products from Yunnan Province. The health risks of these species should be evaluated further and can be mitigated by adopting modern fermentation technologies. With the increasing urbanization and labor shortages in China, mechanized fermentation is becoming more prevalent. Industrialized fermentation offers advantages in safety, efficiency, and consistency and typically employs selected microbial starter cultures. The yeast cultures isolated here are valuable for developing safe and efficient fermentation technologies for traditional foods.

Multiple yeast species were typically involved in the alcoholic beverages examined (Suppl. material 1: table S1). Besides *S.cerevisiae*, the dominant species *P.kudriavzevii* and *Wickerhamomycesanomalus* are thought to contribute to flavor compound formation, such as organic acids and ethyl esters (Song et al. 2024). These beverages are usually initiated using amylolytic starters (jiuqu), and the dominant species, *Saccharomycopsisfibuligera*, detected in these starters, is an amylolytic yeast crucial for starch breakdown (Wang et al. 2021). Fermented milk products showed the highest yeast species diversity. While it is common to find *K.marxianus*, *Y.lipolytica*, and *S.cerevisiae* in milk fermentation, the roles of frequently isolated species such as *Candidazeylanoides*, *Clavisporalusitaniae*, *Diutinarugosa*, *Torulasporadelbrueckii*, and *Rhodotorulamucilaginosa* remain to be determined. The presence of the first three species—known opportunistic pathogens—raises health concerns.

In the past, new yeast species were mostly isolated from plants, and only limited new species were found from fermented foods (Boekhout et al. 2021). Guo et al. (2019) isolated *Halobasidiumxiangyangense*, a new xylose-utilizing yeast, from the pickling sauce used to make Datoucai in Xiangyang, Hubei, China. Liu et al. (2018) isolated *Yarrowiabrassicae* from traditional Chinese sauerkraut in Nanyang, Henan. Sukrita et al. (2022) isolated *Kazachstaniasurinensis* from *Pla-som* (fermented fish) in Ubon Ratchathani, Surin, and Sisaket in the northeast part of Thailand. The discovery of the five new yeast species from traditional fermented foods in this study implies more unknown yeast species remain to be discovered from such previously well-studied substrates and niches. The functions of the five new species in food fermentation are unclear. They were not among the dominant species in the samples from which they were isolated. They probably originated from surrounding environments or raw materials of the fermented foods concerned. However, the role of *Wickerhamiellashiruii* sp. nov. in food fermentation is worthy of further study, especially considering the fact that *W.shiruii* sp. nov. and the other *Wickerhamiella* species are usually unable to ferment sugars. Though only one strain (CGMCC 2.7449) of the new species was isolated from a traditional rice wine sample collected in this study, 16 additional strains belonging to this new species were previously isolated from different countries. Interestingly, 13 of the non-Chinese strains were isolated from dairy or fermented dairy products in different countries. It will be interesting to study the mechanism underlying the adaptation of this novel species to fermentation environments.

## ﻿Conclusion

Through a citizen science initiative combined with on-site investigation and sampling by the authors, we collected 255 samples of traditional fermented foods from ethnic minority areas in China. The sampled foods covered alcoholic beverages, amylolytic starters, fruit vinegar, and fermented products of milk, vegetables, cereals, legumes, fish, meat, and other materials. A total of 516 yeast strains belonging to 81 yeast species, including four new species, were isolated from these samples. The new yeast species are formally described as *Blastobotrysguizhouensis* sp. nov., *Wickerhamiellashiruii* sp. nov., *Trichosporonjiuqu* sp. nov., and *Parajaminaeaalba* sp. nov. *Saccharomycescerevisiae*, *Pichiakudriavzevii*, *Wickerhamomycesanomalus*, *Saccharomycopsisfibuligera*, and *Clavisporalusitaniae* are the dominant yeast species isolated, proving their significant role in the food fermentation process. The result illustrates the high diversity of yeasts in indigenous fermented foods in ethnic minority areas in China and calls for a more extensive and systematic microbiological study of traditional fermented foods in remote and underdeveloped areas not only in China but also around the world. The outcome of the research will enrich the collection and preservation of microbial resources with special values and will also be helpful for the improvement of the quality and safety of traditionally fermented foods.

## Supplementary Material

XML Treatment for Blastobotrys guizhouensis

XML Treatment for Wickerhamiella shiruii

XML Treatment for Trichosporon jiuqu

XML Treatment for Parajaminaea alba

## ﻿Additional information

### Use of AI

No use of AI was reported.

### Adherence to national and international regulations

All the fungal strains used in this study have been legally obtained, respecting the Convention on Biological Diversity (Rio Convention).

### Author ORCIDs

Shuang Hu https://orcid.org/0000-0001-7285-7626

Qi-Yang Zhu https://orcid.org/0009-0008-1708-1085

Hai-Yan Zhu https://orcid.org/0000-0001-5547-4922

Jun-Yu Liu https://orcid.org/0009-0002-8912-5127

Yue Shi https://orcid.org/0009-0001-1099-9677

Yan-Jie Qiu https://orcid.org/0009-0008-8397-9766

Zhang Wen https://orcid.org/0000-0002-7400-0391

Ai-Hua Li https://orcid.org/0000-0003-0584-3720

Pei-Jie Han https://orcid.org/0000-0001-8342-739X

Feng-Yan Bai https://orcid.org/0000-0001-9823-6430

### Data availability

All gene and genome sequences generated in this study are available in GenBank under the accession numbers provided, with all additional data included in the manuscript and its supplements.

## Supplementary materials

Supplementary material 1 Additional tables Data type xlsx Explanation note **table S1.** The fermented foods collected from ethnic minority areas of China in this study and the yeast species isolated from each sample. **table S2.** Distribution of yeast species in different types of fermented food sampled. **table S3.** Comparisons of physiological and biochemical characters of the new species with their closely related species. **table S4.** The ITS and D1/D2 sequences, genome data, and source of all strains used in this study. **table S5.** Whole-genome average nucleotide identity (ANI) values of *Wickerhamiella*, *Blastobotrys*, and *Trichosporon* species. https://binary.pensoft.net/file/1370376This dataset is made available under the Open Database License (http://opendatacommons.org/licenses/odbl/1.0/). The Open Database License (ODbL) is a license agreement intended to allow users to freely share, modify, and use this Dataset while maintaining this same freedom for others, provided that the original source and author(s) are credited.Shuang Hu

Supplementary material 2 Additional figures Data type pdf Explanation note **fig. S1.** Representative traditional fermented foods of ethnic minorities of China collected in this study. **fig. S2.** Neighbor‐joining phylogenetic tree showing the phylogenetic position of *W.shiruii* sp. nov. based on the D1/D2 sequence. **fig. S3.** Neighbor‐joining phylogenetic tree showing the phylogenetic position of *W.shiruii* sp. nov. based on the ITS sequence. **fig. S4.** Red cells correspond to 95% or more tetra correlation. **fig. S5.** Heatmap of tetranucleotide frequency correlation coefficients (tetra) of 27 *Blastobotrys* and three *Trichomonascus* species with available genome sequences. **fig. S6.** Heatmap of tetranucleotide frequency correlation coefficients (tetra) of eight *Trichosporon* and one *Cutaneotrichosporon* species with available genome sequences. https://binary.pensoft.net/file/1370377This dataset is made available under the Open Database License (http://opendatacommons.org/licenses/odbl/1.0/). The Open Database License (ODbL) is a license agreement intended to allow users to freely share, modify, and use this Dataset while maintaining this same freedom for others, provided that the original source and author(s) are credited.Shuang Hu
