# Supplementary figures and images for: ﻿Yeast diversity in traditional fermented foods of ethnic minorities in China, with the descriptions of four new yeast species
Source: IMA Fungus. 2025 Jul 9;16:e146163. doi: 10.3897/imafungus.16.146163 (PMC12268275; doi:10.3897/imafungus.16.146163)

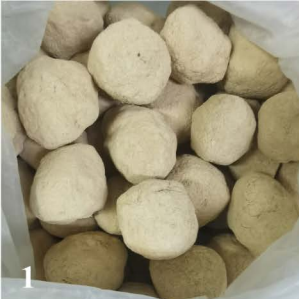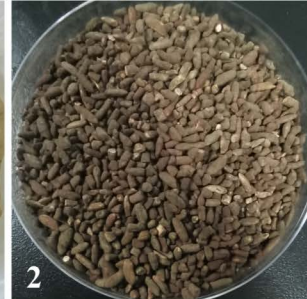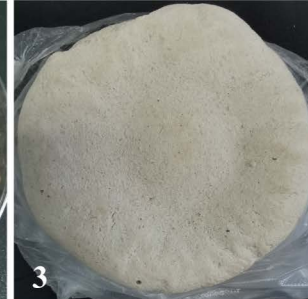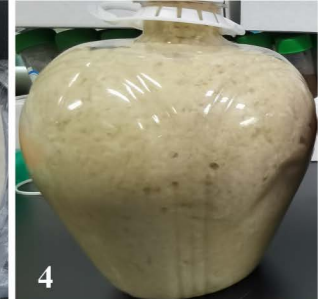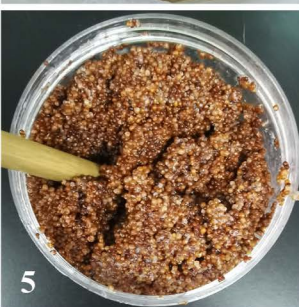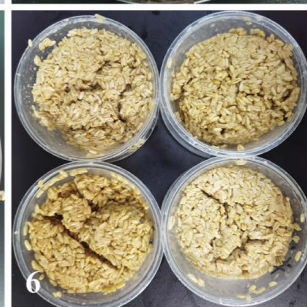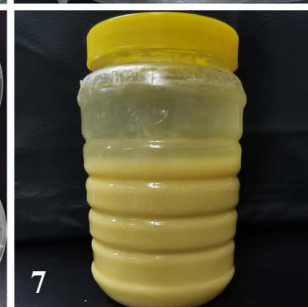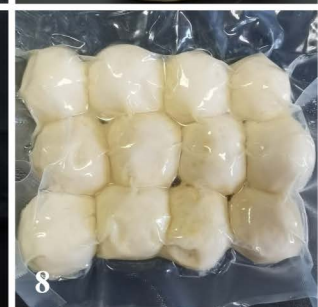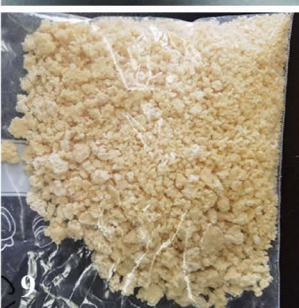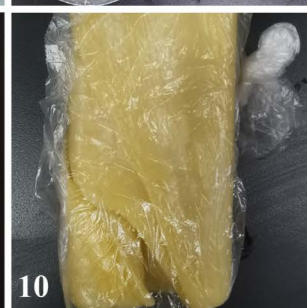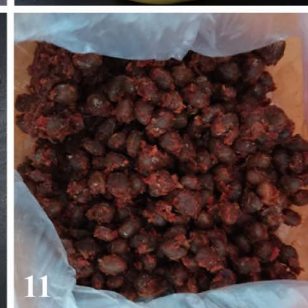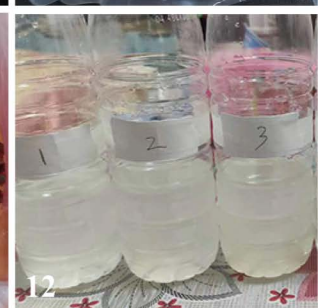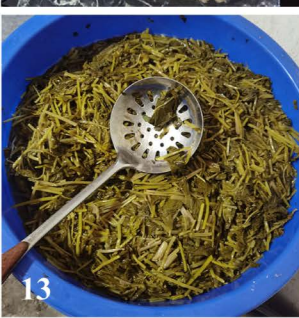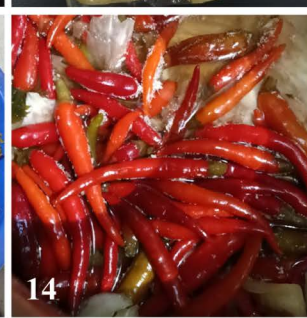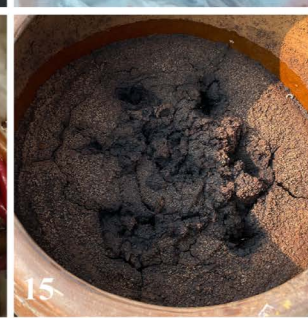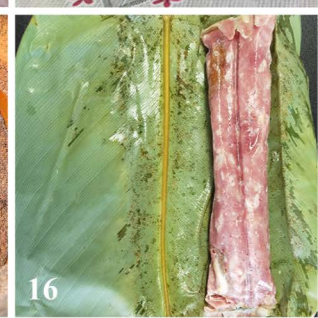

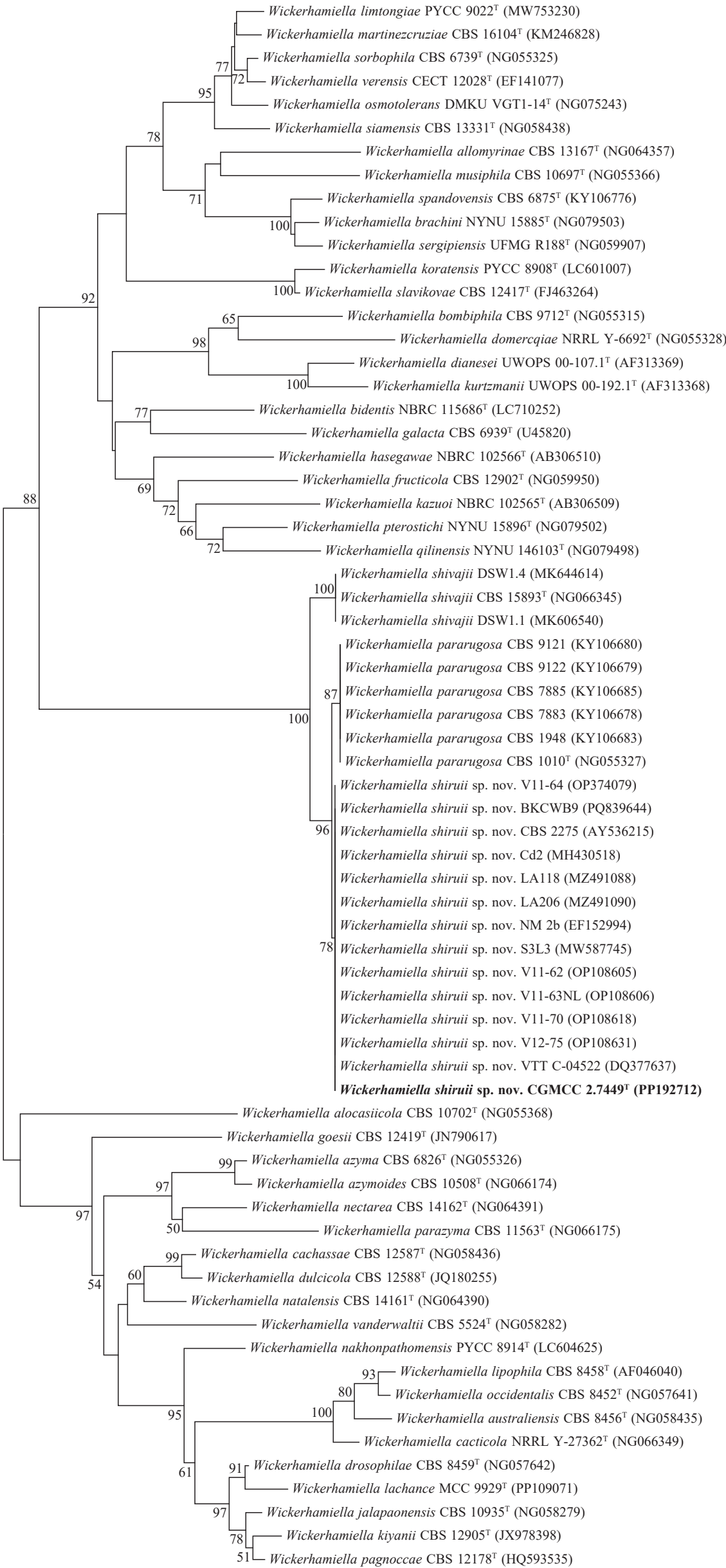

0.02

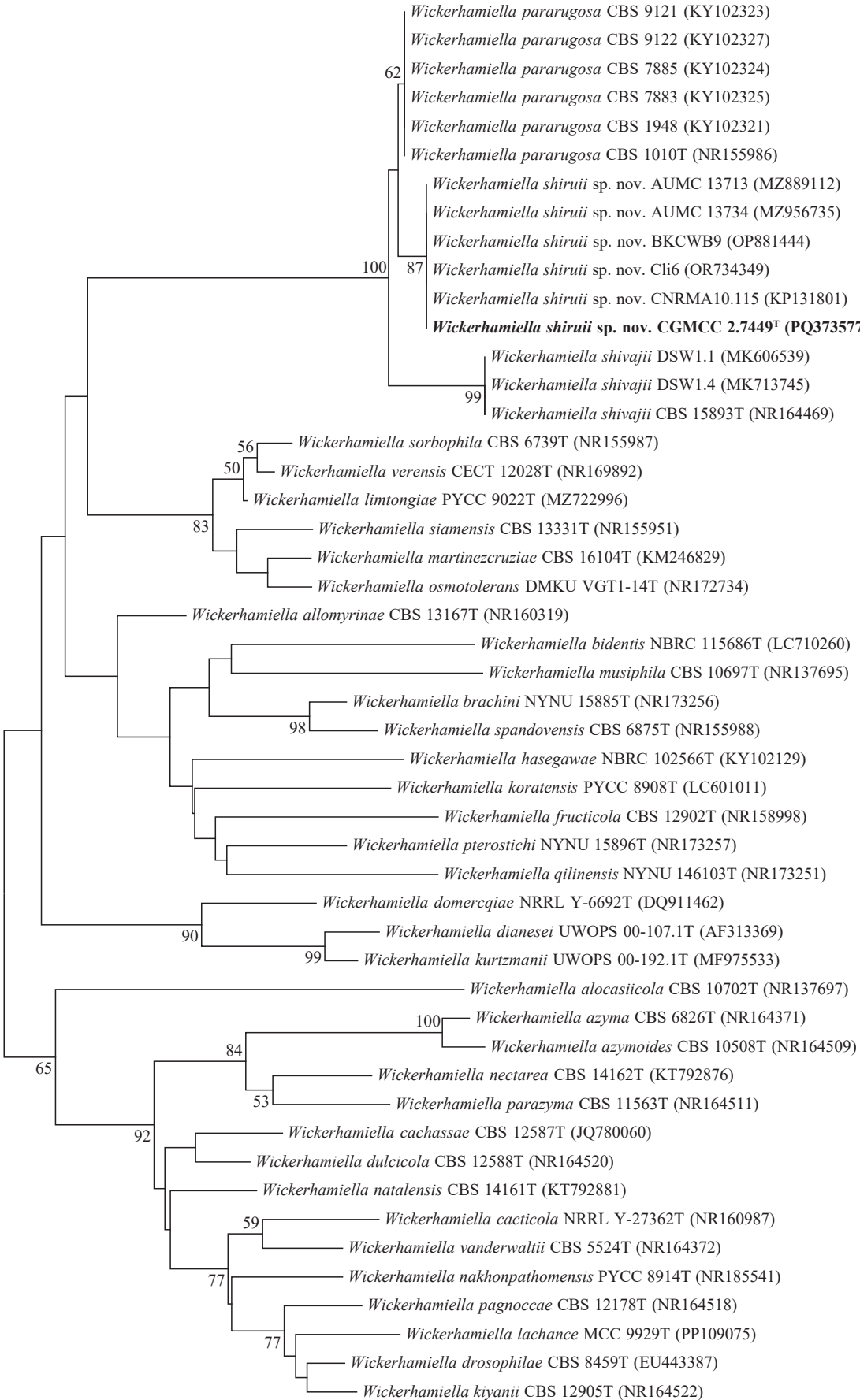

0.02

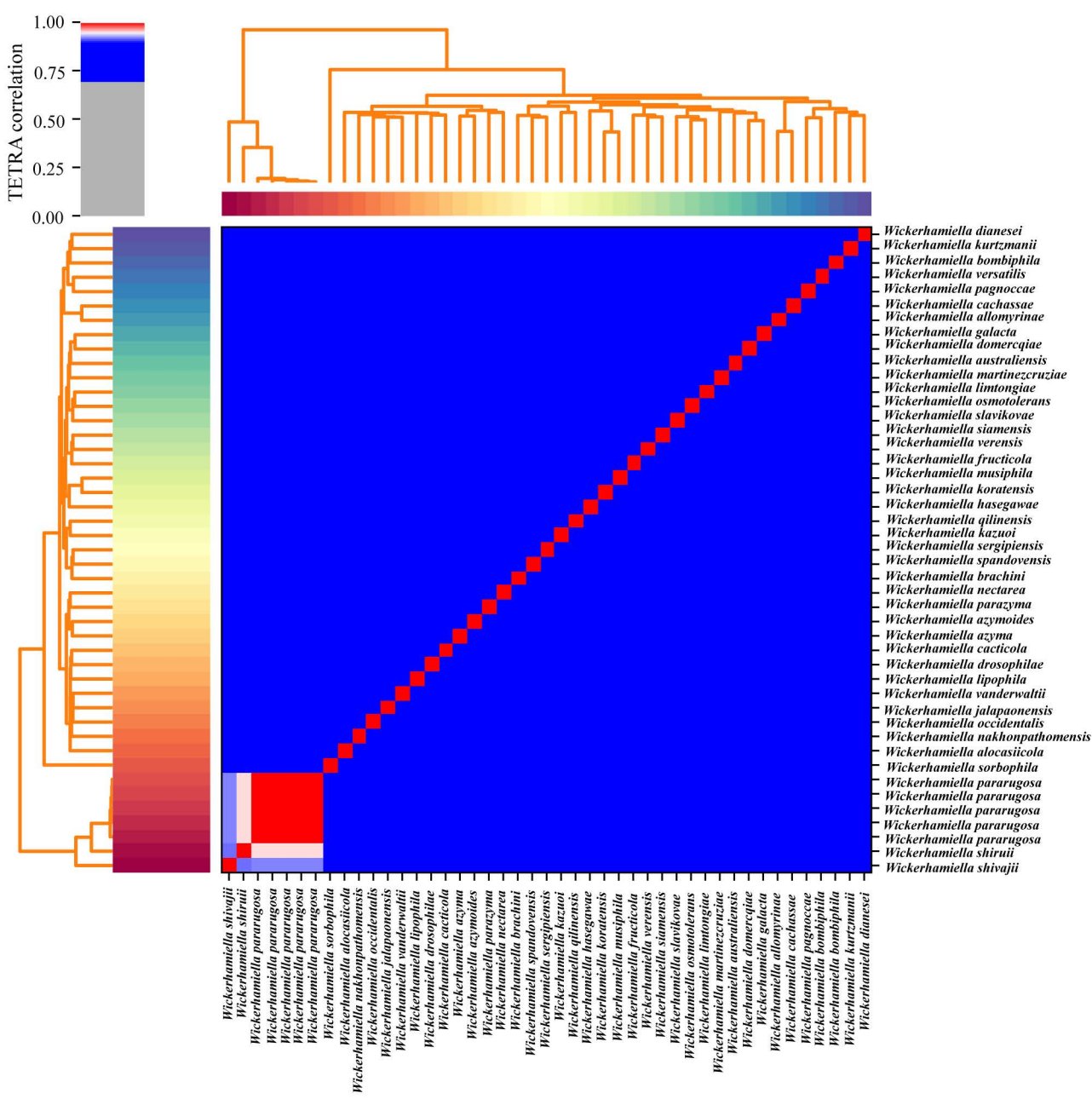

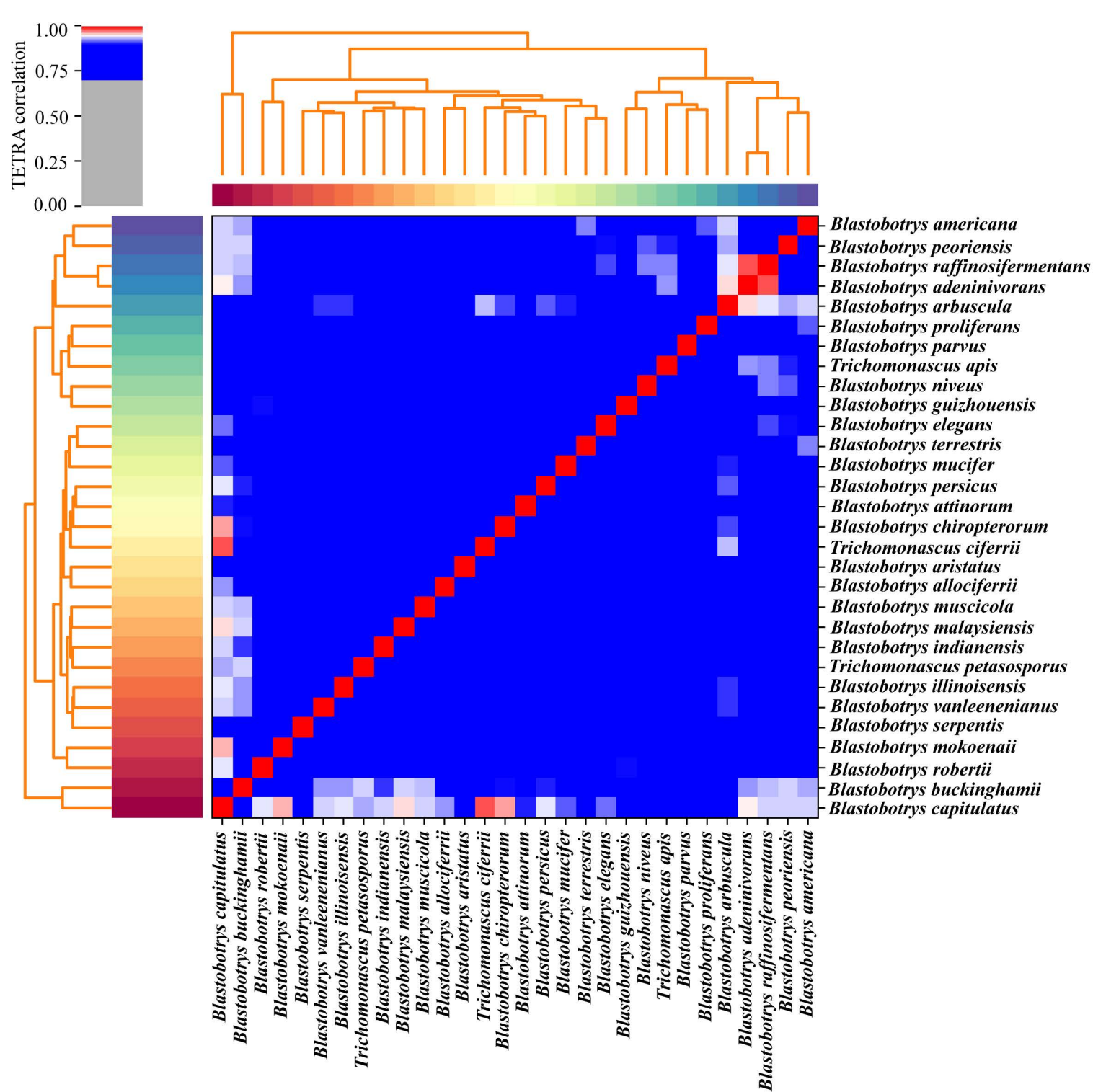

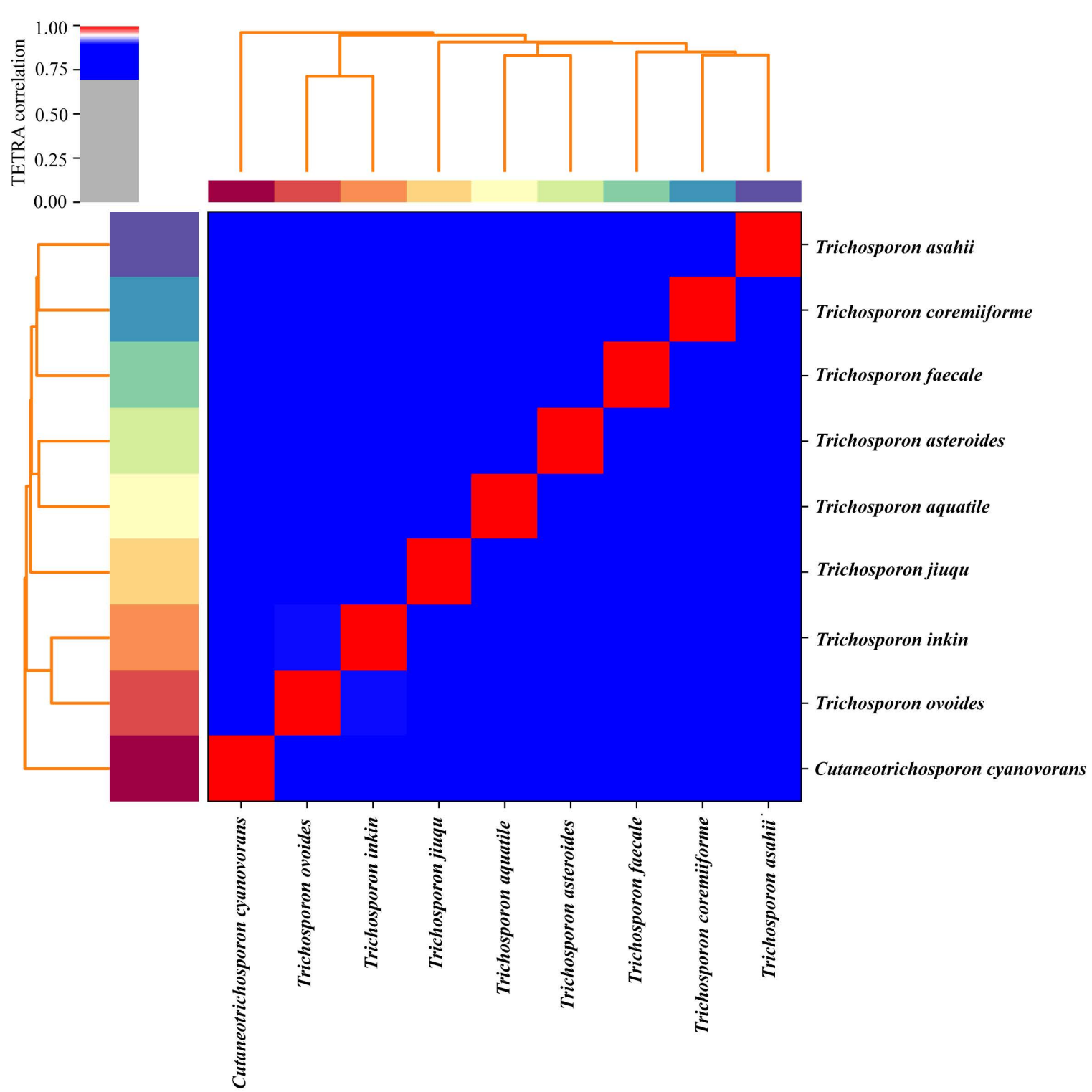

Supplement: Supplementary material 2 — Additional figures [file imafungus-16-e146163-s002.pdf]
